# Effects of Mutations on Replicative Fitness and Major Histocompatibility Complex Class I Binding Affinity Are Among the Determinants Underlying Cytotoxic-T-Lymphocyte Escape of HIV-1 Gag Epitopes

**DOI:** 10.1128/mBio.01050-17

**Published:** 2017-11-28

**Authors:** Yushen Du, Tian-Hao Zhang, Lei Dai, Xiaojuan Zheng, Aleksandr M. Gorin, John Oishi, Ting-Ting Wu, Janice M. Yoshizawa, Xinmin Li, Otto O. Yang, Otoniel Martinez-Maza, Roger Detels, Ren Sun

**Affiliations:** a Cancer Institute, Collaborative Innovation Center for Diagnosis and Treatment of Infectious Diseases, School of Medicine, Zhejiang University, Hangzhou, China; b Department of Molecular and Medical Pharmacology, University of California, Los Angeles, Los Angeles, California, USA; c Molecular Biology Institute, University of California, Los Angeles, Los Angeles, California, USA; d Key Laboratory of Animal Virology of Ministry of Agriculture, Zhejiang University, Hangzhou, China; e Department of Microbiology, Immunology & Molecular Genetics, David Geffen School of Medicine, University of California, Los Angeles, Los Angeles, California, USA; f Department of Epidemiology, Jonathan and Karin Fielding School of Public Health, University of California, Los Angeles, Los Angeles, California, USA; g Department of Pathology and Laboratory Medicine, David Geffen School of Medicine, University of California, Los Angeles, Los Angeles, California, USA; h Department of Medicine. David Geffen School of Medicine, Division of Infectious Diseases, University of California, Los Angeles, Los Angeles, California, USA; i AIDS Healthcare Foundation, Los Angeles, California, USA; j Department of Obstetrics and Gynecology, David Geffen School of Medicine, University of California, Los Angeles, Los Angeles, California, USA; Medical School, University of Athens

**Keywords:** CTL escape, Gag epitopes, HIV-I, high-throughput fitness profiling, MHC binding prediction, intrapatient viral evolution

## Abstract

Certain “protective” major histocompatibility complex class I (MHC-I) alleles, such as B*57 and B*27, are associated with long-term control of HIV-1 *in vivo* mediated by the CD8^+^ cytotoxic-T-lymphocyte (CTL) response. However, the mechanism of such superior protection is not fully understood. Here we combined high-throughput fitness profiling of mutations in HIV-1 Gag, *in silico* prediction of MHC-peptide binding affinity, and analysis of intraperson virus evolution to systematically compare differences with respect to CTL escape mutations between epitopes targeted by protective MHC-I alleles and those targeted by nonprotective MHC-I alleles. We observed that the effects of mutations on both viral replication and MHC-I binding affinity are among the determinants of CTL escape. Mutations in Gag epitopes presented by protective MHC-I alleles are associated with significantly higher fitness cost and lower reductions in binding affinity with respect to MHC-I. A linear regression model accounting for the effect of mutations on both viral replicative capacity and MHC-I binding can explain the protective efficacy of MHC-I alleles. Finally, we found a consistent pattern in the evolution of Gag epitopes in long-term nonprogressors versus progressors. Overall, our results suggest that certain protective MHC-I alleles allow superior control of HIV-1 by targeting epitopes where mutations typically incur high fitness costs and small reductions in MHC-I binding affinity.

## IMPORTANCE

Understanding the mechanism of viral control achieved in long-term nonprogressors with protective HLA alleles provides insights for developing functional cure of HIV infection. Through the characterization of CTL escape mutations in infected persons, previous researchers hypothesized that protective alleles target epitopes where escape mutations significantly reduce viral replicative capacity. However, these studies were usually limited to a few mutations observed *in vivo*. Here we utilized our recently developed high-throughput fitness profiling method to quantitatively measure the fitness of mutations across the entirety of HIV-1 Gag. The data enabled us to integrate the results with *in silico* prediction of MHC-peptide binding affinity and analysis of intraperson virus evolution to systematically determine the differences in CTL escape mutations between epitopes targeted by protective HLA alleles and those targeted by nonprotective HLA alleles. We observed that the effects of Gag epitope mutations on HIV replicative fitness and MHC-I binding affinity are among the major determinants of CTL escape.

## INTRODUCTION

HIV-1-specific CD8^+^ cytotoxic T lymphocytes (CTLs) represent the most critical immune response that limits HIV-1 replication *in vivo* (1–3). Their antiviral activity has been demonstrated in laboratory experiments and clinical observations (1, 4–6). HIV-1-specific CTLs from infected persons show robust killing of HIV-1-infected cells *in vitro* (7). In macaque models, *in vivo* depletion of CTLs causes consistent increase of viremia (3). CTLs recognize HIV-1-infected cells through the binding of short, virus-derived peptide epitopes that are presented on the cell surface via major histocompatibility complex class I (MHC-I) molecules. Although CTLs play an essential protective role, they ultimately fail to control HIV replication and to prevent disease progression in most individuals. By mutation, HIV-1 can escape from CTL killing in multiple ways, such as reduction of the binding of viral epitopes to MHC-I, interference with epitope processing, and impairment of binding recognition by T cell receptor (8–11). The pressure for HIV-1 to escape CTL recognition is in fact a major driver of viral evolution at the individual and population levels (12–14).

The genotype of MHC-I alleles determines the HIV-1 epitopes available for presentation and thus the targeting of CTLs (11, 15). Certain “protective” alleles are enriched in long-term nonprogressors (LTNPs) and are associated with superior viral immune control. However, the mechanism behind this protection is not fully understood (15–20). One proposed hypothesis is that CTL escape mutations in epitopes presented by these alleles significantly reduce viral replicative capacity. In support of this hypothesis, the high fitness costs of some CTL escape mutations have been investigated and observed experimentally (21–24). However, previous studies have relied on the identification of epitope mutations in samples from infected persons and characterization of their phenotypes *in vitro* and have revealed only a small number of mutations that survived the selection process. Moreover, as virus fitness *in vivo* depends on both its intrinsic replicative capacity and its ability to evade CTL pressure, protective MHC-I alleles may also target epitopes where evasion of CTL recognition is more difficult to occur. Therefore, a systematic examination of the fitness cost and the effect on CTL escape is necessary to obtain a quantitative comparison of epitopes targeted by protective and nonprotective MHC-I alleles (25–29).

In this study, we integrated multiple approaches, including high-throughput fitness profiling, *in silico* prediction of MHC-peptide binding affinity, and analysis of intraperson virus evolution, to systematically determine the differences between epitopes presented by protective MHC-I alleles and those presented by nonprotective MHC-I alleles with respect to HIV-1 Gag mutations. Mutations in epitopes corresponding to protective MHC-I alleles had higher replicative capacity cost and lower levels of reductions in MHC-I binding affinity. The conclusion was supported with consistent differences observed in Gag sequences from HIV-1-infected LTNPs and progressors.

## RESULTS

### High-throughput fitness profiling of HIV-1 Gag mutations.

**FIG 1:**
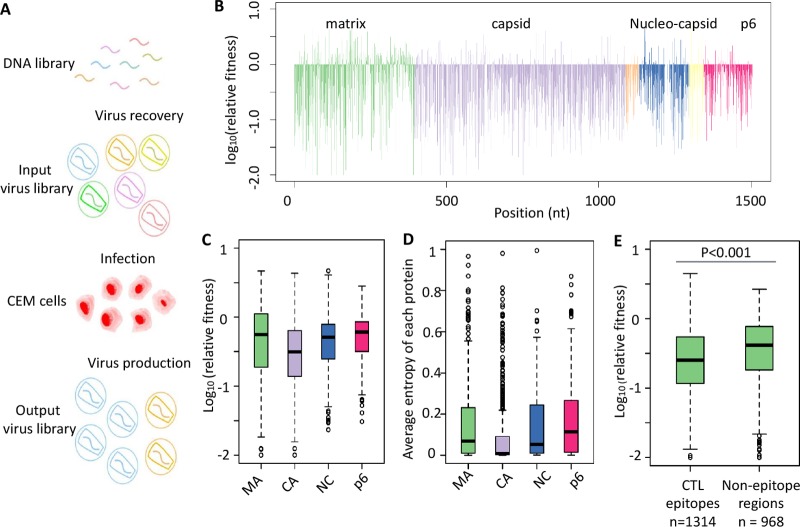
Quantitative high-throughput fitness profiling of HIV-I Gag polyprotein. (A) Experimental design of high-throughput fitness profiling of the HIV-1 Gag region. (B) Relative fitness score of each point mutation in Gag. Each Gag protein is labeled by a different color. MAb, matrix; CA, capsid; SP1, spacer peptide 1; NC, nucleocapsid; SP2, spacer peptide 2; p6, p6 protein; nt, nucleotide. (C) Average relative fitness scores of missense mutations in each Gag protein are shown using a box plot. (D) The average entropy of each Gag protein was calculated based on naturally occurred variants in the HIV sequence database at Los Alamos National Laboratory. (E) Relative fitness scores of mutations within or outside CTL epitope regions were compared. CTL epitopes were defined according to the 2013 update of best-characterized epitopes from the Los Alamos Database. A total of 1,314 mutations within CTL epitopes and a total of 968 mutations outside CTL epitopes were calculated.

We have previously demonstrated the feasibility of using quantitative high-throughput genetics to systematically evaluate the fitness effects of point mutations in HIV-1, HCV and influenza virus (25–27, 29). In this study, we generated plasmid libraries of single nucleotide mutations in the Gag region of HIV-1 molecular clone NL4-3 using error-prone PCR mutagenesis. The corresponding virus libraries were reconstituted in 293T cells by transfecting the plasmid libraries followed by two successive passages of 6 days each in a human leukemic T cell line (CEM) (Fig. 1A). Relative fitness (RF) scores, representing the replicative capacities of individual mutants, were calculated as the ratio of the frequency in the library after the two passages to the frequency in the input viral library (Fig. 1B; see also Table S1 in the supplemental material). The mutant library covered 74% (3,340/4,509) of all possible single nucleotide mutations and 27% (2,788/10,020) of single amino acid mutations in Gag. The clear separation of the RF scores between synonymous mutations and missense mutations suggested efficient selection of viable versus nonviable mutants in the passaging process (see Fig. S1A in the supplemental material). We further quantified the effect of missense mutations on replicative capacity and the fraction of lethal mutations for four major proteins encoded in the Gag region. These four proteins displayed various levels of mutation tolerability (Fig. 1C; see also Fig. S1B). For example, the fitness costs of capsid mutations were significantly higher than those seen with all other proteins (*P* < 0.001, *P* < 0.001, and *P* < 0.001 compared with matrix, nucleocapsid, and p6, respectively; two-tailed Wilcoxon rank sum test). Capsid also had more lethal mutations than other proteins (*P* = 0.007, *P* < 0.001, and *P* < 0.001 compared with matrix, nucleocapsid, and p6, respectively; two-tailed Fisher exact test). Around 20% of the missense mutations on capsid were lethal for viral replication in our profiling (30). The fitness effects of mutations in individual Gag protein correlated well with genetic diversity in naturally occurring sequences in the Los Alamos National Laboratory HIV Sequence Database (https://www.hiv.lanl.gov/content/index) (Fig. 1D; see also Fig. S1C and D).

10.1128/mBio.01050-17.1 FIG S1  High-throughput fitness profiling and sequence diversity of clinical isolates. (A) Relative fitness distributions of synonymous and missense mutations for capsid protein. Similar distributions were obtained for other proteins in Gag. (B) Fraction of lethal mutations in missense mutations. Lethal mutations were defined as the mutations with relative fitness scores below −0.95, which represented the bottom 2.5% of synonymous mutations. MAb, matrix; CA, capsid; SP1, spacer peptide 1; NC, nucleocapsid; SP2, spacer peptide 2; p6 protein. Data represent results of a two-tailed Fisher exact test. (C and D) Correlation between relative fitness scores and sequence diversity of natural isolates. The dashed trend line represents LOESS regression. Nucleotide positions were grouped evenly by their Shannon diversity data. Group1 had diversity values of ~0.001 to ~0.006. Group 2 had diversity values of ~0.006 to ~0.033. Group 3 had diversity values of ~0.033 to ~0.184. Group 4 had diversity values of ~0.184 and more. The groups had 293, 287, 311, and 287 nucleotide positions, respectively. Error bars represent standard errors. Download FIG S1, TIF file, 2.6 MB. Copyright © 2017 Du et al. 2017 Du et al. This content is distributed under the terms of the Creative Commons Attribution 4.0 International license.

10.1128/mBio.01050-17.6 TABLE S1  Fitness profiling of Gag mutations. The relative fitness scores (RF scores) of single nucleotide mutations in Gag region are listed. Download TABLE S1, XLSX file, 0.1 MB. Copyright © 2017 Du et al. 2017 Du et al. This content is distributed under the terms of the Creative Commons Attribution 4.0 International license.

As Gag-specific CTLs are important for viral control (1, 21, 31), we examined whether mutations in previously characterized CTL epitopes carry higher fitness cost than in other regions. CTL epitopes were defined according to the 2013 update of best-characterized epitopes from the Los Alamos National Laboratory HIV Immunology Database (https://www.hiv.lanl.gov/content/index). Our fitness profiling of Gag covered 1,314 missense mutations in CTL epitopes and 968 missense mutations in nonepitope regions. Mutations in epitopes had higher fitness cost than those in nonepitope regions (Fig. 1E; two-tailed *t* test, *P* < 0.001), suggesting that Gag-specific CTLs target regions of the proteins with lower tolerance of mutations. Moreover, 70% (50/72) of the epitopes were located on capsid, which was the most conserved protein in the Gag region. Overall, our comprehensive data support the idea that the intolerance of mutations in Gag epitopes might be one of the reasons for better viral control by Gag-specific CTLs.

### Systematic evaluation of effects of Gag epitope mutations on MHC-I binding affinity.

One of the proposed mechanisms whereby HIV-1 can escape CTL killing is via epitope mutations that reduce their binding affinity to MHC-I (8, 32). Several data-driven computation programs have been developed to predict affinity of peptide binding to specific MHC-I molecules (33–35) and thereby allow identification of epitope variants that facilitate escape from CTL recognition. NetMHC is the state-of-art predictor and is based on an artificial neural network (34, 35), achieving up to 80% correlation with experimental data (36). We used netMHC-4.0 to estimate the effects of all single amino acid mutations in Gag CTL epitopes (compared to NL4-3 as the index sequence) on the binding affinity (dissociation constant [*K*_*d*_]) to MHC-I (Table S2). A total of 62 epitopes were included in the analysis (excluding epitopes with predicted *K*_*d*_ > 10 μM).

10.1128/mBio.01050-17.7 TABLE S2  Summarized information for epitopes. The position, corresponding MHC-I types, fitness scores, and binding affinity data are listed for all Gag epitopes. The ones included in the linear regression model (Fig. 3D) are marked in the last column. Download TABLE S2, XLSX file, 0.02 MB. Copyright © 2017 Du et al. 2017 Du et al. This content is distributed under the terms of the Creative Commons Attribution 4.0 International license.

**FIG 2:**
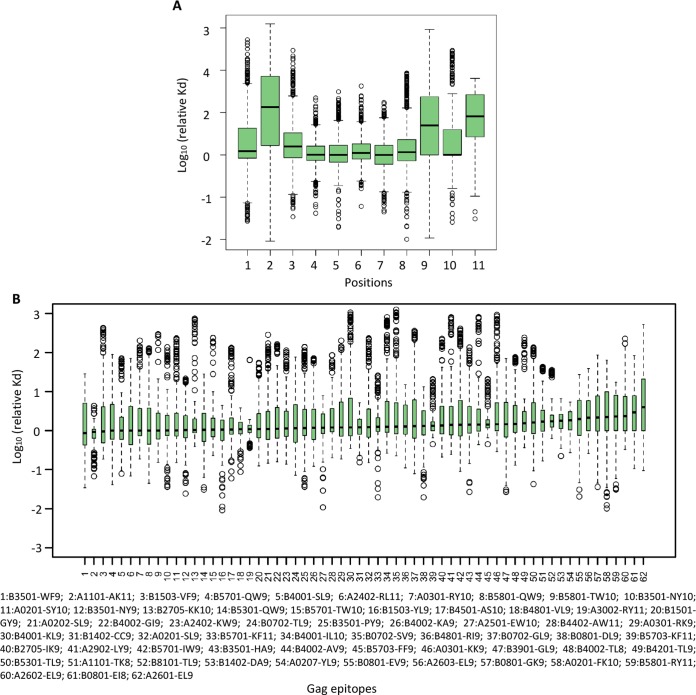
Systematic evaluation of effects of mutations on MHC-I binding affinity. (A) Effects on MHC-I binding affinity of single amino acid mutations at different positions within an epitope. The binding affinity of mutated epitopes was predicted by the use of netMHC-4.0. (B) The distribution of predicted changes in MHC-I binding affinity caused by single amino acid mutations across CTL epitopes in Gag (~171 to ~209 mutations for each epitope, depending on the length).

Peptides bind to MHC-I primarily through anchor residues, which are usually located at position 2 and the C terminus (9th or 11th residue according to the length of peptide) of the peptide. We thus examined the effect of mutations at different positions within an epitope. For each position on an epitope, the missense mutations across all 62 epitopes were examined. A total of 11,580 mutations were included in the analysis. As expected, the greatest drop of binding affinity was observed at anchor residues (2nd, 9th, and 11th residue; *P* < 0.001 when comparing each of the anchor residues with other residues; two-tailed Wilcoxon rank sum test) Fig. 2A, validating the accuracy of using netMHC4.0 to predict binding affinity.

Next, we compared the effects of mutations on MHC-I binding affinity across different Gag epitopes (Fig. 2B; see also Fig. S2A). Notably, different Gag epitopes showed various profiles of changes of binding affinity caused by single amino acid mutations. For example, most mutations in epitopes targeted by HLA B*2705 remained robust, with fewer mutations leading to increased *K*_*d*_ (i.e., lower binding affinity), suggesting that evasion of CTL recognition via reduced binding to MHC-I is uncommon; in contrast, epitopes targeted by HLA A*0207 were more sensitive to mutations. By comparing the *in silico* binding affinity prediction with the fitness profiling of mutations in each epitope, we further evaluated the relationship between MHC-I binding affinity and viral replicative capacity (RF scores). A weak but significant negative correlation was observed (Fig. S2B, rho = −0.093, *P* = 0.003), which may reflect a tradeoff between viral fitness and the reduction of MHC-I binding affinity for escape (32).

10.1128/mBio.01050-17.2 FIG S2  MHC-I binding affinity change of different epitopes. (A) Distribution of the average changes of MHC-I binding affinity caused by single mutations in epitope regions. (B) A weak negative correlation between relative fitness scores and binding affinity drop for all missense mutations in epitope regions was seen. Download FIG S2, TIF file, 2.6 MB. Copyright © 2017 Du et al. 2017 Du et al. This content is distributed under the terms of the Creative Commons Attribution 4.0 International license.

### Impacts of Gag epitope mutations on viral replication and MHC-I binding for epitopes presented by protective versus nonprotective MHC-I alleles.

The replicative capacity of HIV-1 and its ability to escape CTL recognition are two possible determinants of viral replication *in vivo*. To explore the mechanism of superior viral control observed in individuals with protective MHC-I alleles, we combined fitness profiling of mutations and prediction on MHC-I binding affinity to examine the difference between the CTL epitopes targeted by protective MHC-I alleles and those targeted by nonprotective MHC-I alleles.

**FIG 3:**
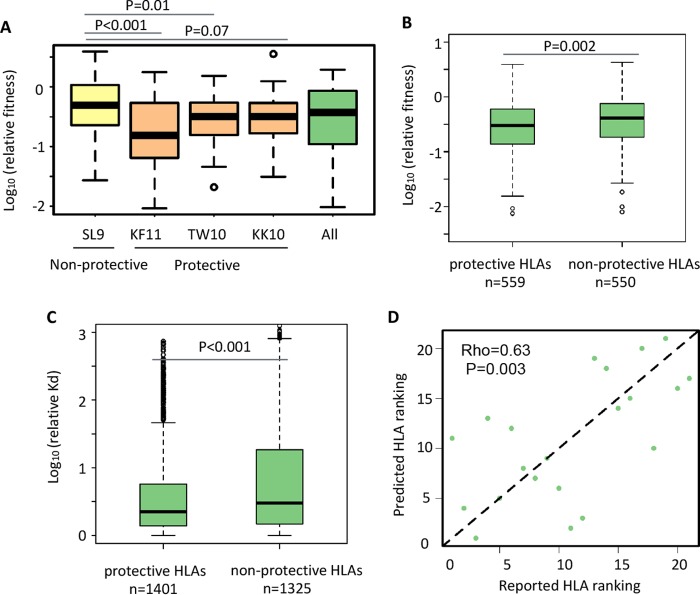
Systematic analysis of differences between protective and nonprotective MHC-I alleles. (A) Relative fitness scores of mutations in indicated epitopes. Epitopes shown as examples are SL9 (Gag positions 77 to 85), KF11 (Gag positions 163 to 173), TW10 (Gag positions 240 to 249), and KK10 (Gag positions 263 to 272). (B) Relative fitness scores of mutations in epitopes presented by protective or nonprotective MHC-I alleles. A total of 559 mutations in the epitopes targeted by protective MHC-I alleles and a total of 550 mutations targeted by nonprotective MHC-I alleles were analyzed. (C) MHC-I binding affinity changes of mutations in epitopes presented by protective or nonprotective MHC-I alleles were compared. A total of 1,401 mutations in the epitopes targeted by protective MHC-I alleles and a total of 1,325 mutations in the epitopes targeted by nonprotective MHC-I alleles were analyzed. (D) The correlation between predicted ranking of MHC-I protectiveness and ranking calculated based on TIHIVC study data. Predicted ranking was fitted by a linear regression model using the average effect of mutations on relative fitness scores and MHC-I binding affinity as variables (Spearman correlation, 0.63; *P* = 0.003).

We first examined several well-characterized epitopes presented by the protective HLA alleles B*57 (KF11 [position 162 to 172] and TW10 [position 240 to 249]) and B*27 (KK10 [position 263 to 272]) and observed that mutations in these epitopes led to greater loss of viral fitness than in the epitopes presented by the nonprotective HLA allele A*02 (SL9 [position 77 to 83]) (Fig. 3A) (37, 38). Furthermore, we ranked MHC-I alleles by the ratio of their prevalence in HIV controllers to their prevalence in progressors based on The International HIV Controllers (TIHIVC) study (Table S3) (2). The top 5 MHC-I alleles with available binding affinity predictions (B*5701, B*2705, B*1402, B*2501, and B*5801) were assigned as “protective,” while the bottom 5 (A*3002, B*0702, A*2902, B*3501, and B*4001) were assigned as “nonprotective.” We observed that the mutations in Gag epitopes presented by protective MHC-I alleles showed significantly greater loss of RF scores than the mutations presented by nonprotective alleles (*P* = 0.004; two-tailed *t* test) (Fig. 3B). These results suggest that protective MHC-I alleles promote targeting HIV-1 epitopes that are less tolerant of mutations.

10.1128/mBio.01050-17.8 TABLE S3  Ranking of MHC-I types. The frequency of occurrence of each MHC-I type in HIV controllers and progressors is shown according to the TIHIVC study data. The ranking of MHC-I types was calculated based the ratio of the corresponding prevalence in HIV controllers to that in progressors. Download TABLE S3, XLSX file, 0.01 MB. Copyright © 2017 Du et al. 2017 Du et al. This content is distributed under the terms of the Creative Commons Attribution 4.0 International license.

Next, we examined the effect of mutations on the MHC-I binding affinity of epitopes presented by protective versus nonprotective MHC-I alleles. Interestingly, single amino acid mutations in epitopes presented by protective alleles showed lower levels of reduction in binding affinity to MHC-I (*P* < 0.001; two-tailed *t* test) (Fig. 3C). Overall, these data suggest that the epitopes presented by protective MHC-I alleles have two important properties: higher fitness costs and less abrogation of MHC-I binding caused by mutations in the epitope sequences. These results were robust if considering B*2705 and B*5701 only as protective MHC-I alleles and reached statistical significance in the TIHIVC study (Fig. S3).

10.1128/mBio.01050-17.3 FIG S3  Comparison of B*5701 and B*2705 with other MHC-I types. (A) Relative fitness scores of mutations in epitopes presented by B*5701 and B*2705 or nonprotective MHC-I alleles. (B) MHC-I binding affinity changes of mutations in epitopes presented by B*5701 and B*2705 or nonprotective MHC-I alleles were compared. (C) Relative fitness scores of mutations in epitopes presented by B*5701 and B*2705 or other MHC-I alleles. (D) Changes of MHC-I binding affinity of mutations in epitopes presented by B*5701 and B*2705 or other MHC-I alleles were compared. Data represent results of a two-tailed Wilcoxon rank sum test. Download FIG S3, TIF file, 2.6 MB. Copyright © 2017 Du et al. 2017 Du et al. This content is distributed under the terms of the Creative Commons Attribution 4.0 International license.

We further examined whether these two properties of CTL epitopes (fitness cost and MHC-I binding affinity) might explain the protective effect of different MHC-I alleles. We first tested if a single property is sufficient to explain the protectiveness of MHC-I. Average relative fitness scores alone were correlated only weakly with the ranking of protectiveness of MHC-I alleles, while the effects of mutations on MHC-I binding did not show a significant correlation with the ranking of their protective effect (Fig. S4). Then, we examined whether combining the two properties can better explain the protectiveness. Indeed, the ranking of the MHC-I types fitted by a linear regression model that includes both properties as predictor variables was significantly correlated with the actual ranking (Spearman rank rho = 0.63, *P* = 0.003) (Fig. 3D). Consistent with the comparison between protective and nonprotective MHC-I alleles (Fig. 3B and C), this finding suggests that these two variables could largely account for their influence on immune containment of HIV-1.

10.1128/mBio.01050-17.4 FIG S4  Predicted MHC-I ranking by relative fitness scores or MHC-I binding affinity. (A) MHC-I protectiveness ranking was predicted by the relative fitness scores of missense mutations in targeted epitope regions. A weak correlation was observed between reported MHC-I protectiveness rankings and predicted rankings. (B) MHC-I protectiveness ranking was predicted by the MHC-I binding affinity change of missense mutations in the targeted epitope regions. No significant correlation was observed between reported MHC-I protectiveness rankings and predicted rankings. Download FIG S4, TIF file, 2.6 MB. Copyright © 2017 Du et al. 2017 Du et al. This content is distributed under the terms of the Creative Commons Attribution 4.0 International license.

### Mutations in HIV-1 Gag observed in intraperson viral evolution.

**FIG 4:**
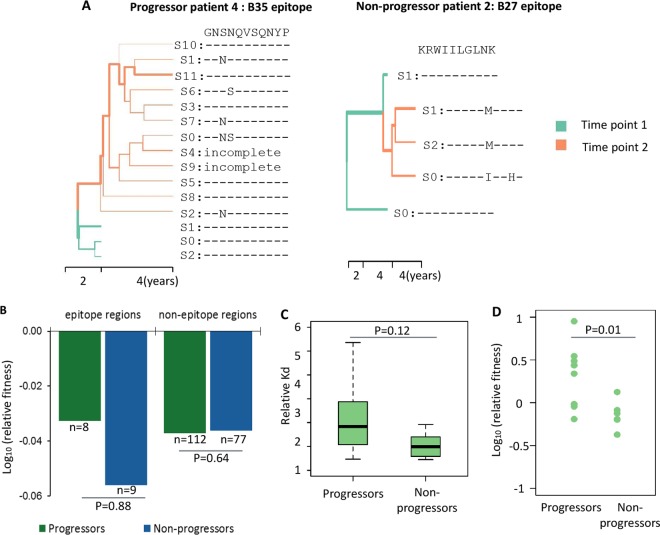
HIV intraperson evolution in LTNPs and progressors. (A) Representative phylogenetic trees of virus haplotypes of LTNPs and progressors. Viral haplotypes were assembled by PredictHaplo. Maximum clade credibility (MCC) trees were constructed by BEAST. The mutations in a representative HLA epitope are labeled. The width of branches is proportional to the abundance of the corresponding haplotypes in the population. The colors green and orange represent sampling time point 1 and time point 2. (B) Relative fitness scores of naturally aroused variants in both groups. (C) Predicated effects of epitope mutations in samples on MHC-I binding affinity. For each individual, the epitopes were selected based on the corresponding HLA serotypes. Epitope sequences from all reconstructed viral haplotypes (11 from progressors and 6 from LTNP) were included in the analysis. (D) Relative fitness scores of possible MHC-1 escape mutations in both groups. Possible MHC-I escape mutations from each individual were defined as the ones with levels of binding affinity lower than those seen with the global consensus sequences.

Finally, we examined the evolution of HIV-1 epitopes in 4 progressors and 4 long-term nonprogressors (LTNPs) with chronic HIV-1 infection. Samples were collected from the Multicenter AIDS Cohort Study (MACS) (Table S4) with matched CD4 cell percentages at the first time point. All subjects were antiretroviral therapy (ART) naïve; thus, the major selective pressure for viral evolution was imposed by the host immune system. Progressors proceeded to AIDS-related death, while LTNPs maintained stable (drop of less than 10%) blood CD4^+^ T cell levels (Table S4) over 4 years of observation after enrollment in the cohort. Proviral DNA was extracted from 10 million peripheral blood mononuclear cells (PBMC), and the entire *gag* region (1,500 bp) was amplified and subjected to deep sequencing (Materials and Methods and Fig. S5A). The consensus *gag* sequences examined at enrollment and 4 years later displayed the expected phylogenetic clustering of sequences in each individual (Fig. S5B). We also reconstructed full-length (1,500-bp) viral haplotypes by the use of PredictHaplo. The frequency of mutations calculated from reconstructed haplotypes was highly correlated with the frequency of mutations in the raw data, indicating that the reconstruction of haplotypes was reliable (Fig. S4C). For both groups, we observed an accumulation of mutations in specific HLA epitopes (example shown in Fig. 4A) and a minor increase in the Shannon entropy of epitope regions at the second time point (Fig. S4D), suggesting that these epitope regions might be under the control of positive selection to escape CTL restriction in the infected individuals.

10.1128/mBio.01050-17.5 FIG S5  HIV intraperson evolution revealed by sequencing. (A) The sequencing depth of each sample from infected individuals. (B) A phylogenetic tree was constructed using the consensus sequence of each person’s sample at each time point. (C) The frequency of mutations in the reconstructed viral haplotypes is highly correlated with the frequency in the raw data. (D) Sequence diversity of epitope regions for progressors and LTNPs at the two time points. Download FIG S5, TIF file, 2.6 MB. Copyright © 2017 Du et al. 2017 Du et al. This content is distributed under the terms of the Creative Commons Attribution 4.0 International license.

10.1128/mBio.01050-17.9 TABLE S4  Information of progressors and LTNPs. Information representing results from progressors and LTNPs that were included in our sequence analysis (Fig. 4) is shown. Download TABLE S4, XLSX file, 0.01 MB. Copyright © 2017 Du et al. 2017 Du et al. This content is distributed under the terms of the Creative Commons Attribution 4.0 International license.

We next examined the effect of the observed mutations on viral replicative fitness and CTL escape. Mutations on virus in each individual were determined by comparing the sequences seen at time point 2 to the consensus sequence at time point 1. The fitness effects of these mutations were extracted from our profiling data. For CTL epitope regions, we found that the mutations observed in LTNPs had slightly higher fitness cost than those in progressors (Fig. 4B), although the results did not reach statistical significance. Additionally, the CTL epitope mutations observed in progressors resulted in a more substantial drop in the binding affinity to MHC-I than the mutations in LTNPs (Fig. 4C). Finally, we extracted the possible MHC-I escape mutations from each individual by comparing the binding affinity of mutations with the global consensus defined in the database at Los Alamos National Laboratory (39). The escape mutations associated with LTNPs showed significantly lower replicative fitness than those associated with progressors (*P* = 0.01; two-tailed Wilcoxon tank sum test) (Fig. 4D). Thus, we observed the same trend as that seen in our previous analysis, where LTNPs are linked with higher replicative fitness cost and lower drops in binding affinity to MHC-I introduced by mutations in corresponding epitopes.

## DISCUSSION

In the present study, by combining high-throughput fitness profiling and *in silico* prediction of MHC-peptide binding affinity, we observed that epitopes targeted by protective MHC-I alleles have two important properties: mutations in these epitopes are more deleterious with respect to viral replication and have a smaller effect on evasion of CTL recognition than those targeted by nonprotective MHC-I alleles. Collectively, these two properties can largely account for the superior viral control conferred by protective MHC-I alleles. We observed a similar trend in virus populations in infected individuals, wherein escape mutations in LTNPs were associated with higher fitness costs and smaller effects on HLA binding affinity than were seen in escape mutations in progressors.

CTL escape mutations with highly predictable patterns are frequently observed in HIV-infected individuals (8, 9, 12, 15, 17, 33, 40–42). Previous studies have revealed that escape mutations in epitopes presented by protective MHC-I alleles often result in a high cost with respect to replicative capacity or require the preexistence of compensatory mutations (11, 32, 40, 43). However, those studies were usually restricted to studying a few mutations observed *in vivo*. In this work, we overcame the restrictions represented by the limited sampling with a systematic unbiased fitness profiling of Gag mutations. Consistent with previous observations, our comprehensive fitness data and integrated analyses support the notion that protective MHC-I alleles result in viral epitopes with lower mutational tolerance. Reduced HIV replication capacity resulting from deleterious mutations is associated with reduced rates of CD4 decline and disease progression (11, 16, 44). Thus, the high fitness cost of CTL escape mutations can in part explain the superior viral control observed in individuals with protective MHC-I alleles.

The viral growth that occurs under conditions of CTL selective pressure depends not only on intrinsic replication capacity but also on the ability to escape CTL recognition. Reduction of MHC-I binding affinity through the activity of epitope mutations is another contributor to CTL escape. Based on the calculations performed using NetMHC4.0, we noted that mutations in epitopes presented by protective MHC-I alleles had a smaller effect on MHC-I binding than those presented by nonprotective MHC-I. Furthermore, we found that incorporating the effect of mutations on MHC-I binding into a linear regression model substantially improved the prediction of the protectiveness of MHC-I alleles, in comparison with the model that accounted only for fitness costs.

There are a few caveats pertaining the current study. First, we used random mutagenesis to introduce mutations in the Gag region. Although we optimized the mutation rate to approach 1 mutation per fragment, it is possible that there were multiple mutations in single viral clones, which might bias the fitness score of some mutations. Second, our mutant library was limited to single nucleotide mutations in the NL4-3 virus backbone; thus, we were unable to assess the fitness effect of compensatory mutations or the effect of these mutations on different genetic backgrounds (i.e., different strains of HIV-1). Third, we did not generate every possible amino acid variant at every position in Gag since our mutagenesis method usually introduces single nucleotide changes, thus limiting the diversity of amino acid changes at each position. Fourth, although drops in MHC-I binding affinity are considered to represent a major mechanism of HIV CTL escape (8), other factors such as intracellular epitope processing and recognition of T cell receptor may also contribute to CTL escape. Lastly, the sample size of infected individuals without any antiviral treatment was small for our intraperson viral evolution analysis, which limited the statistical power of our comparisons between LTNPs and progressors in terms of fitness cost and MHC binding. More samples are required for the further validation of our results. However, despite these limitations, we have performed a most comprehensive profiling of variants within Gag, which enables us to integrate with prediction of MHC binding affinity and obtain verification with viral sequence analyses of sequential samples from infected individuals.

Understanding the mechanism of viral control achieved in LTNPs with protective MHC-I alleles provides insights for developing functional cure and T cell-mediated vaccine against HIV (16, 44, 45). Several T cell vaccine strategies focus on using evolutionarily conserved regions in HIV genome as immunogens, with the promise that escape mutations in the conserved regions will incur higher fitness cost (46–50). However, many studies have documented that conserved regions are not necessarily essential for viral fitness, although there is some correlation (27, 29). Our systematic investigations of single amino acid mutations in Gag will more precisely pinpoint the sequences that are essential for viral replication and that are less likely to escape CTL, thereby aiding the rational design of immunogens for vaccine development.

## MATERIALS AND METHODS

### Construction of high-density mutant libraries for the HIV Gag region.

To generate mutant plasmid libraries, we divided the entire *gag* genes in replication-competent proviral plasmid NL43 into 3 fragments, each spanning position 790 to position 1419, position 1419 to position 1995, and position 1995 to position 2292 on HXB2 coordinates. Mutations were randomly introduced into each fragment by performing error-prone PCR using Mutazyme II DNA polymerase (Stratagene). Mutated segments were then ligated back into the proviral backbone. The ligated products were electroporated into high-efficiency MegaX DH10B T1R electrocompetent cells (Invitrogen). Clones (*n* = 5 to 10) were randomly picked and analyzed by Sanger sequencing to estimate the mutation rate (approximately 1 mutation/clone). Approximately 50,000 bacterial colonies were collected for each of the three small libraries.

### Transfection, viral titer determinations, and passage of HIV mutant libraries.

To reconstitute the mutant virus library, approximately 15 million 293T cells were transfected with each mutant plasmid library (one of 3 small libraries; 16 µg) using Lipofectamine 2000 (Life Technologies, Inc.). The cells were rinsed with phosphate-buffered saline (PBS) at 12 to 14 h posttransfection and were maintained in fresh Dulbecco’s modified Eagle’s medium (DMEM) growth media supplemented with 10% fetal bovine serum (FBS) and 1× penicillin-streptomycin. The supernatants were harvested at 72 h posttransfection, filtered through a 0.45-µm-pore-size disposable syringe filter (Olympus), and stored at −80°C in small aliquots. The 50% tissue culture infective dose (TCID_50_) of viral supernatants was measured using GHOST 3-X4/R5 indicator cells (gift of Matthew Marsden and Jerry Zack), which were derived from human osteosarcoma cells and stably transfected with the HIV long terminal repeat (LTR) driving a human green fluorescent protein (hGFP) construct (23). To passage each viral mutant library, approximately 30 million CEM T-lymphocyte cells were used for infection at a low multiplicity of infection (MOI = 0.05) and were supplemented with 2 µg/ml Polybrene (Sigma). At ~14 to ~16 h postinfection, cells were centrifuged at 1,000 rpm for 5 min and washed with PBS followed by the addition of fresh RPMI 1640 growth medium. Extracellular viruses were harvested at approximately 6 days postinfection when syncytium formation can be observed in ~60% to ~80% of cells. Two rounds of passaging were performed for each library.

### Library preparation for deep sequencing.

Viral RNAs were isolated from the viral supernatants using a QIAamp Viral RNA Minikit (Qiagen), treated with DNase I, and reverse transcribed using Superscript III reverse transcriptase (Life Technologies, Inc.). The plasmid mutant libraries or cDNAs from the viral mutant libraries (transfection or infection) were amplified using KOD Hot Start DNA polymerase. The amplified fragments were then ligated with the sequencing adapter, which had three nucleotide multiplexing identifiers (IDs) to distinguish the different samples. An Illumina HiSeq 2000 PE100 system was used for sequencing.

### Sequencing data analysis.

A Burrows-Wheeler aligner (BWA) was used to map sequencing reads to reference sequences (18). Paired-end reads were used for error correction. The relative frequency of each mutation was calculated for each condition, and the relative fitness score was calculated as the difference between the frequency in the passaged library and the frequency in the transfected library. To further improve data quality, mutations with a frequency of <0.01% in the transfection library were filtered out, and possible G-A hypermutations were removed (3). Lethal mutations were defined as the mutations with log_10_(RF score) of less than −0.95. That value represented the distribution peak of nonsense mutations, while only 2.5% of synonymous mutations fell below this cutoff.

### Conservation analysis among clinical isolates.

A total of 6,097 prealigned HIV-1 subtype B Gag sequences were downloaded from the database at Los Alamos National Laboratory. No filter was applied for sampling time, country, or individuals’ information. The Shannon entropy of all residues was calculated by custom scripts, and the resulting data were deposited at https://github.com/Tian-hao/HIV-clinical/.

### Prediction of MHC binding affinity using netMHC4.0.

MHC-I binding affinity (i.e., *K*_*d*_ increase) was calculated by netMHC4.0. Epitope sequences and MHC-I alleles were paired according to the best-characterized HIV-1 CTL epitopes from the database at Los Alamos National Laboratory (51). The binding affinity change is calculated as the ratio of the *K*_*d*_ value determined for the mutated epitope and that determined for the parental epitope with MHC-1.

### Ranking of protectiveness of MHC-I alleles.

We defined the protectiveness of MHC-I alleles as the ratio of the HLA allele prevalence in nonprogressors to the HLA allele prevalence in progressors (2). We then ranked the MHC-I types by two different properties of mutations in the targeting epitopes as follows: (i) rank_fitness_, representing the average effect on viral replication fitness of missense mutations profiled in our fitness data (i.e., relative fitness score), and (ii) rank_binding_, representing the average effect on MHC-I binding affinity of single amino acid substitutions (i.e., increase in *K*_*d*_). We used these two properties of MHC-I types to fit the ranking of protectiveness rank_protect_ using the following linear regression model:

$${\text{rank}}_{\text{protect}}=\alpha \cdot {\text{rank}}_{\text{fitness}}+\beta \cdot {\text{rank}}_{\text{binding}}$$

The best-fit parameters were α = 0.51 and β = 0.35. The fitted ranking of protectiveness showed a better correlation than predictions based solely on rank_fitness_ (see Fig. S3 in the supplemental material), indicating that the effect of mutations on MHC-I binding affinity contributed to the protectiveness of MHC-I alleles.

As a control, we also fitted the ranking of protectiveness using the following alternative model:

$${\text{rank}}_{\text{protect}}=\alpha \cdot {\text{rank}}_{\text{fitness}}+\beta \cdot {\text{rank}}_{\text{random}}$$

where we assigned a trait (rank_fitness_) that was randomly ranked among MHC-I alleles, in addition to the ranking of fitness effects. We found that only 4.3% (43/1,000) randomly ranked traits produced a better fit of rank_protect_ than the incorporated model, suggesting that the effect of mutations on MHC-I binding contributes to the protectiveness of MHC-I alleles.

### Sequencing and analysis of viral samples from infected individuals.

PBMC samples from 4 paired progressors and long-term nonprogressors (LTNPs) were kindly provided from the Multicenter AIDS Cohort Study (MACS). All of these infected individuals were treatment naive and paired according to their CD4 cell percentage at baseline. For each individual, we obtained two PBMC samples at time points 4 years apart, where the first sample was collected at the earliest time point in the MACS cohort. The progressors proceeded to the AIDS phase and died at year 4 during the cohort study, while the LTNPs maintained stable CD4 cell counts. DNAs were extracted from 10 million PBMC from each individual. The entire *gag* region (1,500 bp) was amplified by nested PCR. The gel-purified PCR products were then subjected to random fragmentation by sonication to achieve fragments of 200 to 700 bp. The fragmented libraries were prepared for high-throughput sequencing with an Illumina HiSeq 2000 system.

Viral sequences were mapped onto HIV-1 molecular clone NL4-3. The haplotypes of Gag genes were constructed by the use of PredictHaplo1.0 (52). Consensus sequences were determined. Variations at each nucleotide were identified if a haplotype sequence was found to be different from the consensus sequence. The tree of haplotypes was constructed using Phylip.

A mutation at time point 2 was defined as a single nucleotide polymorphism (SNP) if it was different from the consensus sequence at time point 1. Incomplete reads were filtered out. The epitopes were called for all individuals’ HLA serotypes that had targeted epitopes in the table of the best-defined CTL epitopes. Escape mutations were defined by the ones with a level of MHC-I binding affinity lower than that seen with the global consensus sequence. For prediction of MHC-1 binding affinity, the epitope sequences of all reconstructed haplotypes were used as the input sequences. A total of 59 progressor sequences and a total of 47 LTNP sequences were used. All the custom scripts were deposited at https://github.com/Tian-hao/HIV-clinical/.
