# Supplementary figures and images for: Effects of Mutations on Replicative Fitness and Major Histocompatibility Complex Class I Binding Affinity Are Among the Determinants Underlying Cytotoxic-T-Lymphocyte Escape of HIV-1 Gag Epitopes
Source: mBio. 2017 Nov 28;8(6):e01050-17. doi: 10.1128/mBio.01050-17 (PMC5705913; doi:10.1128/mBio.01050-17)

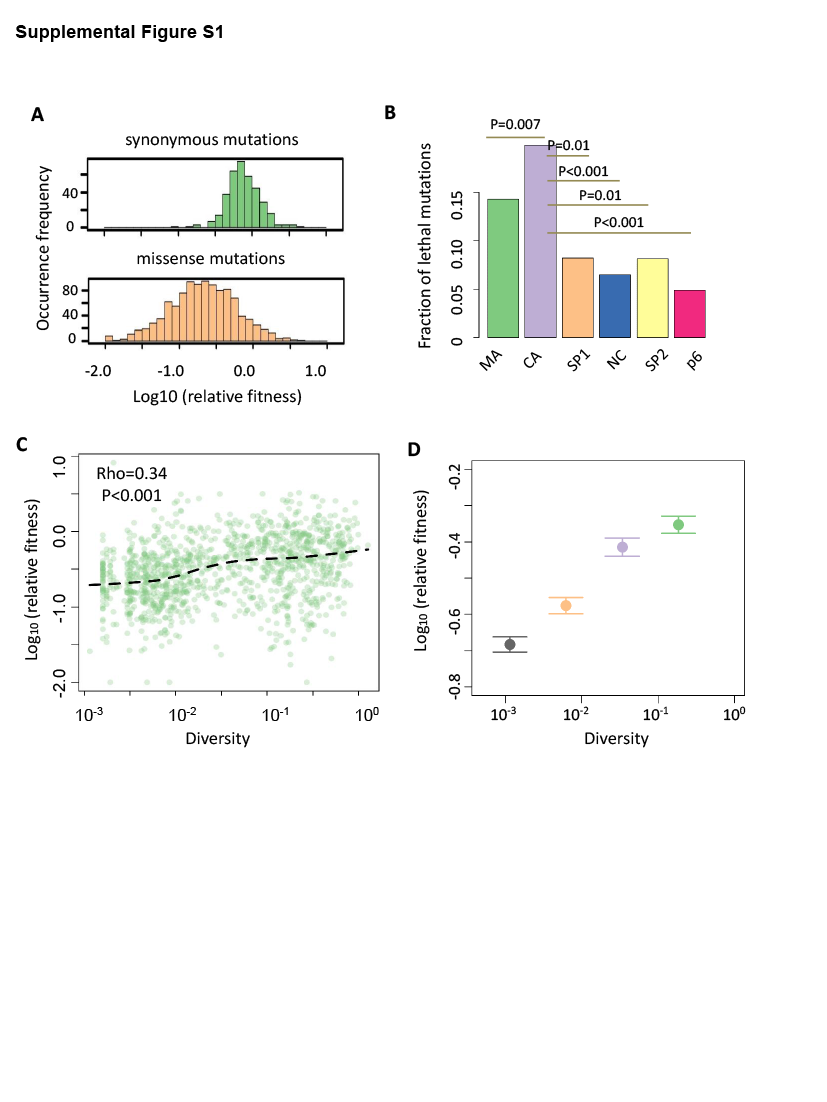

Supplement: FIG S1 [file mbo006173608sf1.tif]

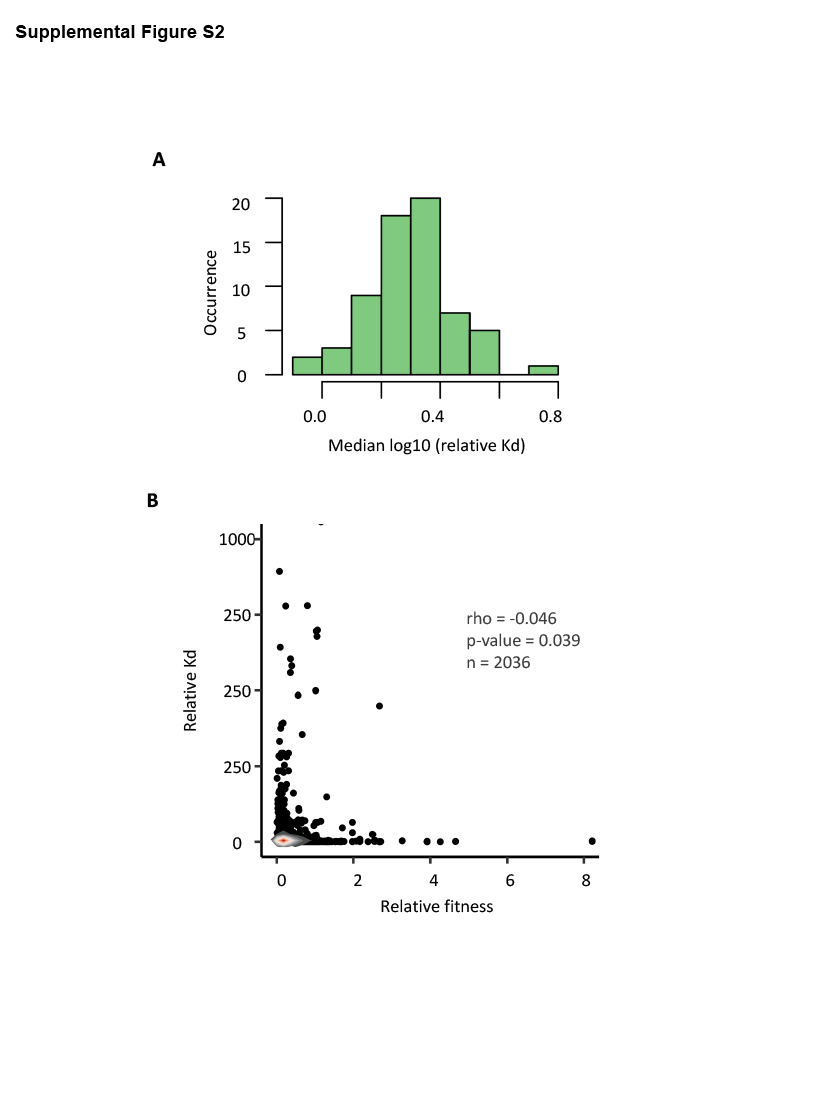

Supplement: FIG S2 [file mbo006173608sf2.tif]

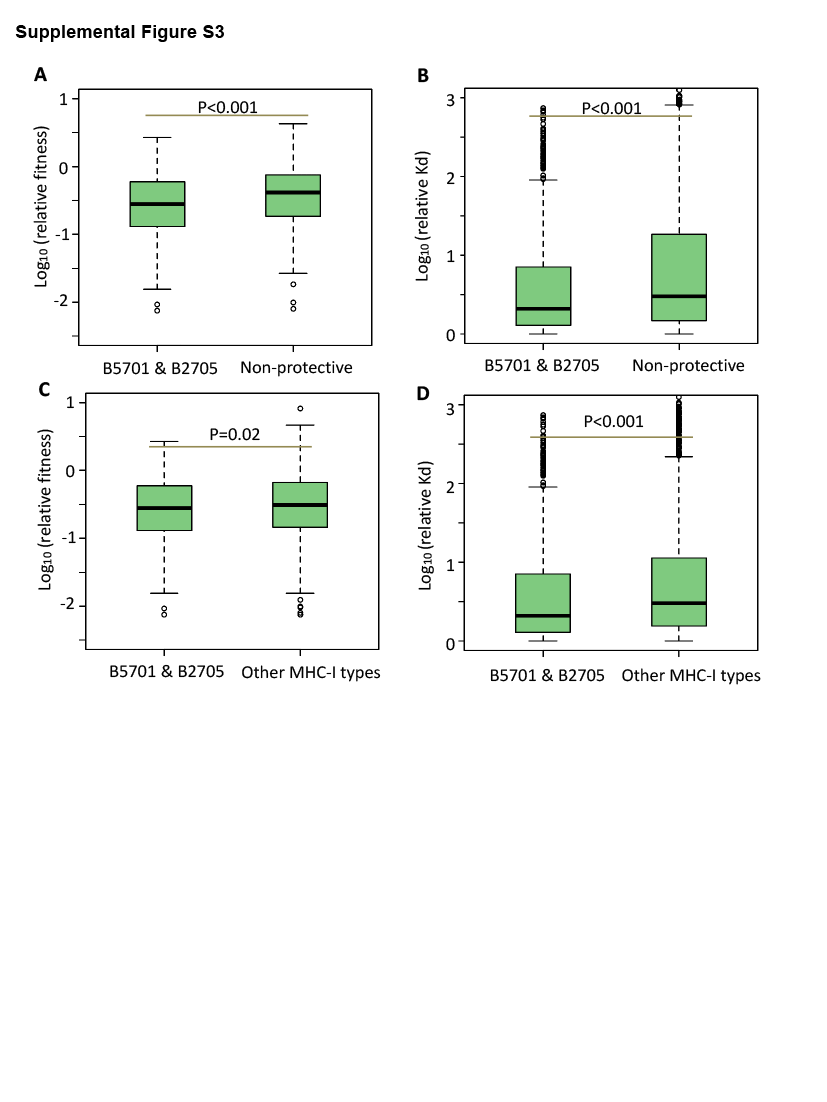

Supplement: FIG S3 [file mbo006173608sf3.tif]

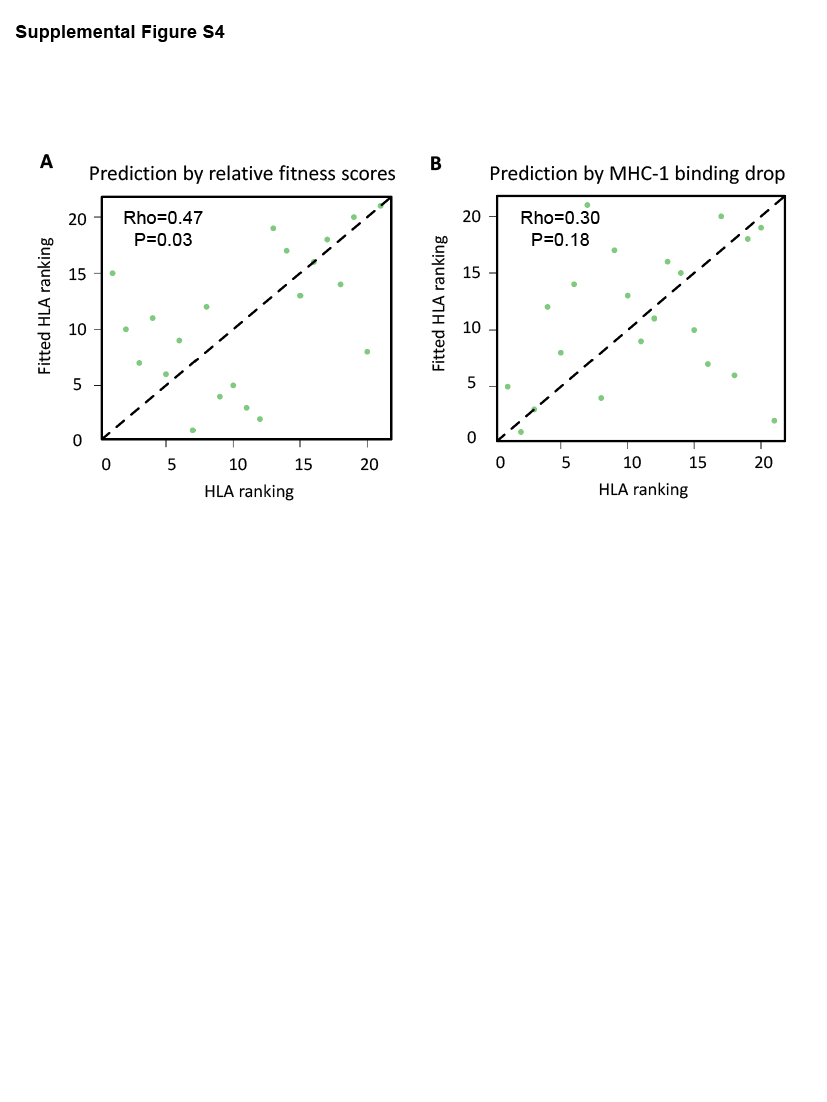

Supplement: FIG S4 [file mbo006173608sf4.tif]

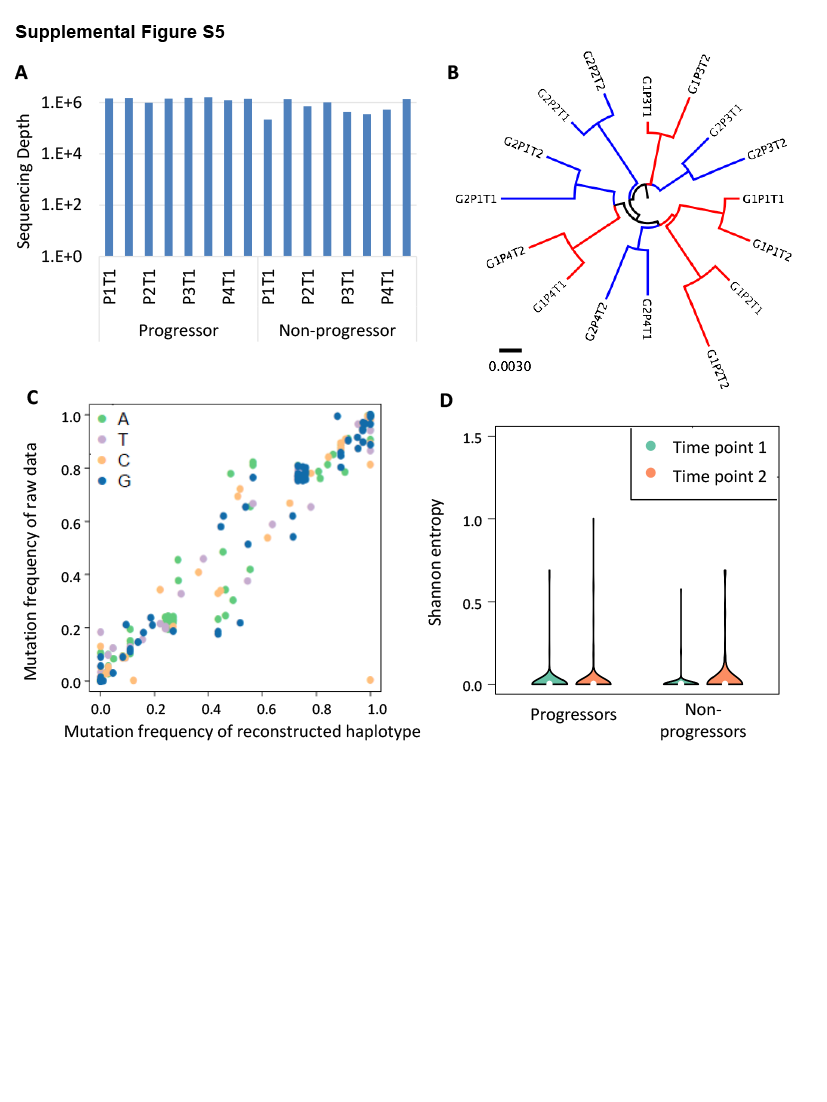

Supplement: FIG S5 [file mbo006173608sf5.tif]
